# Supplementary material for: Divergent auditory activation in relation to inhibition task performance in children and adults
Source: Hum Brain Mapp. 2023 Jul 26;44(15):4972–85. doi: 10.1002/hbm.26418 (PMC10502686; doi:10.1002/hbm.26418)

**
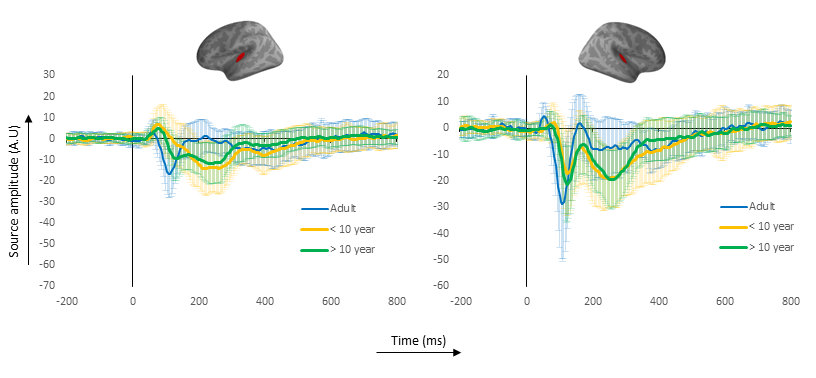
Figure 1.** Age differences in source estimates of the passive listening (PL) auditory responses.


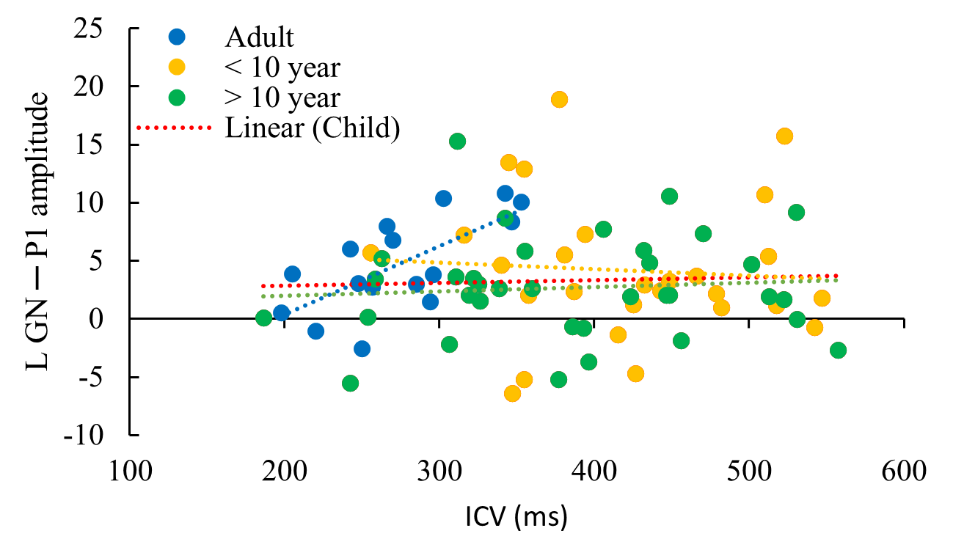


**Figure 2.** Scatterplot of the left hemisphere P1 response to the No-go tone and the intraindividual coefficient of variability (ICV) of the Go/No-go (GN) task of children younger than 10 years (yellow), older than 10 years (green) and adults (blue). The linear trendline of the complete group of children is depicted in red.


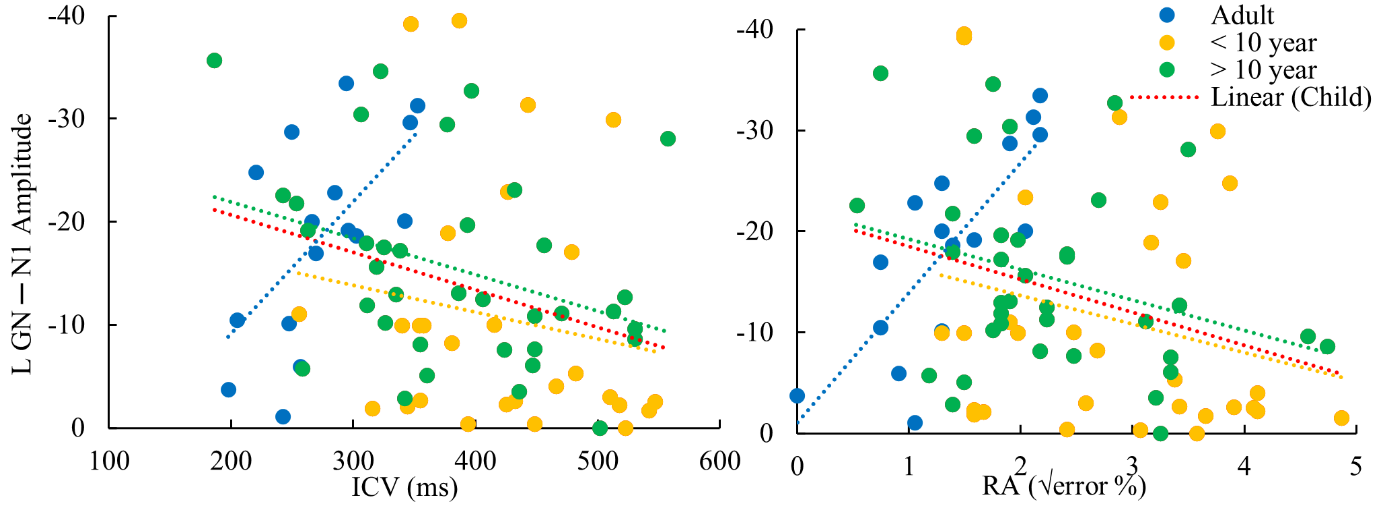
**Figure 3.** Scatterplot of the left hemisphere N1 response to the No-go tone and the intraindividual coefficient of variability (ICV; left) and response accuracy (RA; right) of the Go/No-go (GN) task of children younger than 10 years (yellow), older than 10 years (green) and adults (blue). The linear trendline of the complete group of children is depicted in red.

**Figure 4.** Scatterplot of the left hemisphere P2 response to the No-go tone and the intraindividual coefficient of variability (ICV; left) and Stop-Signal Reaction Time (SSRT; right) of the Go/No-go (GN) task of children younger than 10 years (yellow), older than 10 years (green) and adults (blue). The linear trendline of the complete group of children is depicted in red.
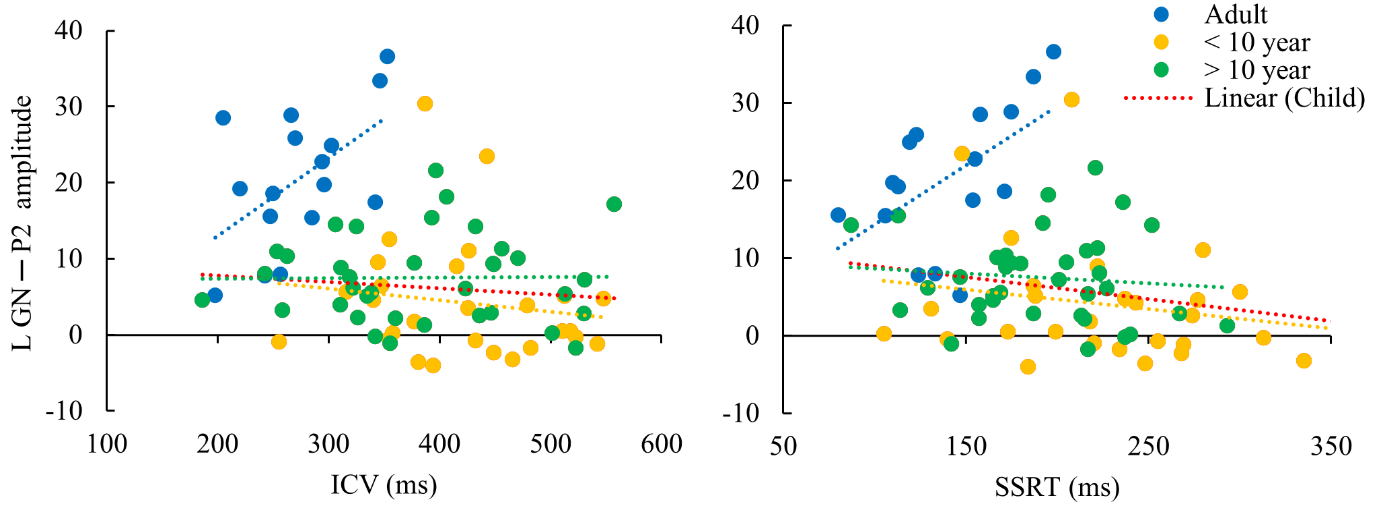

Supplement: Supplementary file 1 — FIGURE S1: Age differences in source estimates of the passive listening (PL) auditory responses. FIGURE S2. Scatterplot of the left hemisphere P1 response to the No‐go tone and the intraindividual coefficient of variability (ICV) of the Go/No‐go (GN) task of children younger than 10 years (yellow), older than 10 years (green) and adults (blue). The linear trendline of the complete group of children is depicted in red. FIGURE S3. Scatterplot of the left hemisphere N1 response to the No‐go tone and the intraindividual coefficient of variability (ICV; left) and response accuracy (RA; right) of the Go/No‐go (GN) task of children younger than 10 years (yellow), older than 10 years (green) and adults (blue). The linear trendline of the complete group of children is depicted in red. FIGURE S4. Scatterplot of the left hemisphere P2 response to the No‐go tone and the intraindividual coefficient of variability (ICV; left) and Stop‐Signal Reaction Time (SSRT; right) of the Go/No‐go (GN) task of children younger than 10 years (yellow), older than 10 years (green) and adults (blue). The linear trendline of the complete group of children is depicted in red. [file HBM-44-4972-s001.docx]
